# Genome Editing Using TALENs in Blind Mexican Cavefish, *Astyanax mexicanus*

**DOI:** 10.1371/journal.pone.0119370

**Published:** 2015-03-16

**Authors:** Li Ma, William R. Jeffery, Jeffrey J. Essner, Johanna E. Kowalko

**Affiliations:** 1 Department of Biology, University of Maryland, College Park, Maryland, United States of America; 2 Genetics, Development and Cell Biology Department, Iowa State University, Ames, Iowa, United States of America; University of Iceland, ICELAND

## Abstract

*Astyanax mexicanus*, a teleost fish that exists in a river-dwelling surface form and multiple cave-dwelling forms, is an excellent system for studying the genetic basis of evolution. Cavefish populations, which independently evolved from surface fish ancestors multiple times, have evolved a number of morphological and behavioral traits. Quantitative trait loci (QTL) analyses have been performed to identify the genetic basis of many of these traits. These studies, combined with recent sequencing of the genome, provide a unique opportunity to identify candidate genes for these cave-specific traits. However, tools to test the requirement of these genes must be established to evaluate the role of candidate genes in generating cave-specific traits. To address this need, we designed transcription activator-like effector nucleases (TALENs) to target two genes that contain coding changes in cavefish relative to surface fish and map to the same location as QTL for pigmentation, *oculocutaneous albinism 2* (*oca2*) and *melanocortin 1 receptor* (*mc1r*). We found that surface fish genes can be mutated using this method. TALEN-induced mutations in *oca2* result in mosaic loss of melanin pigmentation visible as albino patches in F_0_ founder fish, suggesting biallelic gene mutations in F_0_s and allowing us to evaluate the role of this gene in pigmentation. The pigment cells in the albino patches can produce melanin upon treatment with L-DOPA, behaving similarly to pigment cells in albino cavefish and providing additional evidence that *oca2* is the gene within the QTL responsible for albinism in cavefish. This technology has the potential to introduce a powerful tool for studying the role of candidate genes responsible for the evolution of cavefish traits.

## Introduction

Identifying and characterizing causative mutations is crucial to understanding the evolution of traits. In recent years, a substantial amount of work has been done to identify loci responsible for natural variation using genetic mapping techniques (for example [1,2]). A few of these studies have identified the genes likely to be responsible for this phenotypic variation (for example [3,4]). However, a significant challenge that remains is testing for the functional consequences of these candidate genes in the organism itself. Few studies have accomplished this level of analysis (for an exception, see [5]), owing to the lack of genetic tools developed for the organisms of interest.

*Astyanax mexicanus*, a species of teleost fish, is an excellent system for understanding the genetic changes that drive morphological and behavioral evolution. *Astyanax mexicanus* exists in two forms, a river-dwelling surface form and multiple, independently-evolved cave forms (reviewed in [6]). The cave forms of *Astyanax mexicanus* differ from the surface form in a number of ways. Relative to surface fish, cavefish have regressed eyes, a reduction in melanin pigment and an increase in the number and distribution of taste buds and cranial neuromasts, the sensory organs of the lateral line [7–9]. Cavefish behavior is also modified. Cavefish have enhanced feeding behavior and reduced schooling behavior, aggression, and sleep [10–16].

Cavefish and surface fish, which reach sexual maturity around 4–6 months, are interfertile [7], and quantitative trait loci (QTL) mapping studies have been performed on crosses between surface and cavefish to determine the region(s) of the genome associated with many of their phenotypic differences [17–23]. A few of these mapping studies have led to identification of candidate genes and cave-specific genetic lesions within them that are thought to be responsible for changes in cave traits [17,19]. Two of these are pigmentation genes, *melanocortin 1 receptor* (*mc1r*) and *oculocutaneous albinism 2* (*oca2*). The *mc1r* gene is located within a QTL for the reduction in the number of melanophores, the melanin producing pigmentation cells. Two cave populations have alleles of *mc1r* that contain coding changes relative to surface fish, and a morpholino targeting *mc1r* in zebrafish leads to a reduction in melanin pigment that cannot be rescued by coinjection with a cave allele of this gene [19].

Multiple cave populations of *Astyanax mexicanus* are albino and lack all melanin pigmentation. The *oca2* gene lies within the single QTL peak for albinism [17]. The *oca2* alleles from two albino cave populations contain large deletions, and these alleles cannot rescue pigmentation defects in an albino cell line [17]. Furthermore, knockdown of *oca2* by morpholino in surface fish results in albino morphant fish [24]. These studies illustrate the power of QTL mapping in *Astyanax mexicanus* to locate and identify candidate genes and genetic lesions for cave traits. Comparative transcriptional profiling of developing cavefish and surface fish [25,26] and the recent sequencing of the Pachón cavefish genome [27] will aid in the identification of additional candidate genes.

A number of techniques have been used in *Astyanax mexicanus* to evaluate candidate genes for their significance in cavefish evolution, a few of which were highlighted above. These techniques include *in situ* hybridization to look at changes in timing and location of gene expression [28,29], transient overexpression of genes during development [28], and, most recently, transgenic fish have been made to obtain tissue-specific gene expression [30]. Currently, the only option for studying reduced expression of a gene in *Astyanax mexicanus* is by the use of morpholinos [24], short synthetic oligonucleotides that block translation or splicing. While valuable insight has come from morpholino studies on the effects of perturbation of genes during development, morpholinos can result in toxicity and off-target effects [31]. Furthermore, reduction of expression by a morpholino is limited to a few days post fertilization, making morpholinos ineffective for studying adult phenotypes, including most behaviors. For example, it has been suggested that loss of *oca2* is in fact adaptive, resulting in an increase of catecholamines and changes in behavior [24]. Indeed, morpholino knockdown of *oca2* in surface fish resulted in an increase in dopamine levels in larval fish [24]. However, the transient nature of these changes in morpholino-injected fish has made it impossible to study the effects of *oca2* on behavior. It is critical to develop techniques for generating genetic loss of function alleles in *Astyanax mexicanus* to fully understand the role of candidate genes beyond early development.

Transcription activator-like effector nucleases (TALENs) can be used to make targeted mutations at precise locations in the genome. A TALEN consists of a set of repeat-variable diresidues (RVDs) linked to a *FokI* monomer. TALENs target a specific sequence within the genome based on their sequence of RVDs because each RVD binds to a specific nucleotide [32,33]. TALENs can be synthesized artificially and assembled with any order of RVDs to bind to a sequence of interest [32]. Each TALEN is assembled in pairs that bind to adjacent DNA sequences. Once both halves of the TALEN pair bind to the DNA, the *FokI* domains can dimerize and introduce a double-stranded break [34]. These double-stranded breaks can be repaired through non-homologous end joining, which often introduces errors that result in small insertions or deletions (indels). Indels can lead to frameshift mutations in the targeted gene, which can result in the loss of function of the encoded protein. TALENs have been used for genome editing in a number of organisms, including the invertebrates *Drosophila melanogaster* [35] and an annelid *Platynereis dumerilii* [36], and vertebrates *Xenopus laevis* [37], the zebrafish *Danio rerio* [38,39], the catfish *Tachysurus fulvidraco* [40], tilapia [41], and medaka [42].

Here, we use TALENs to introduce mutations into the surface form of *Astyanax mexicanus*. We target two genes that contain coding mutations in cavefish and lie under QTL for pigmentation, *mc1r* and *oca2*. We find that TALENs can be used to mutate these genes in F_0_ founder surface fish and that co-injection of two TALEN pairs can be used to create larger mutations than those obtained using a single TALEN. We show that one out of four founder fish transmitted mutant alleles of *oca2*. Finally, we find that F_0_ fish can be evaluated for pigmentation defects that are likely the result of biallelic mutations in *oca2* in these fish. The future use of this technology will enable us to evaluate the effects of candidate genes in vivo on the evolution of cavefish.

## Materials and Methods

### Animal Husbandry

All animal procedures were in accordance with the guidelines of the National Institutes of Health and were approved by the Institutional Animal Care and Use Committees at Iowa State University and University of Maryland.

Surface fish, cavefish and zebrafish populations were maintained on a 14–10 light-dark cycle. Groups of surface fish were bred by increasing frequency and amount of feeding leading up to the breeding date, and then increasing the temperature by 2°F each night for two nights. Both laboratories have 14 light to 10 hour dark cycles. Using Zeitgeber time where lights are on at ZT0 and off at ZT14, surface fish spawn between ZT15 and ZT19. Zebrafish spawn between ZT0 and ZT3. Eggs were collected in the dark for injection immediately after spawning.

### TALEN design and injection

TALENs were designed using the TAL Effector Nucleotide Targeter 2.0 (https://tale-nt.cac.cornell.edu/node/add/talen) [43,44]. The genomic sequences for *mc1r* and *oca2* were obtained from the cavefish *Astyanax mexicanus* genome (http://useast.ensembl.org/Astyanax_mexicanus/Info/Index). The entire coding sequence of *mc1r*, the *oca2* exon 9 sequence, and the end of intron 20 through the beginning of intron 21 from *oca2* were used to find potential TALEN sites in *Astyanax mexicanus*. The *oca2* exon 9 sequence was used to find TALEN sites in zebrafish. The spacer length was specified as 15 base pairs and the repeat array was specified as 15–20 repeats. TALENs were chosen based on their location and the presence of a restriction enzyme site within the spacer region for genotyping.

The *mc1r* TALEN recognition sequences were: Left TALEN 5’-ACCACAGCATCATGA-3’, right TALEN 5’- AGCACAATAATGGCCA-3’ with a 15-bp spacer 5’- CCACGAGGCGTGCCG-3’ containing a *Bss*SI restriction enzyme site (underlined). The *oca2* TALEN targeting exon 9 recognition sequences were: Left TALEN 5’-GGTCCCTCTCTCGAT-3’, right TALEN 5’-TTCCACCGTTACGTAC-3’ with a 15-bp spacer 5’-GACGCACCAGTCTCT-3’ containing a *Bsr*I restriction enzyme site (underlined). Two TALENs were designed to create an approximately 110-bp deletion around exon 21 in *oca2*. The first TALEN recognition sequences were: Left TALEN 5’-AACATGTTTGTGTTGTT-3’, right TALEN 5’- CCGCCTCTGGCACAG-3’ with a 15-bp spacer 5’-ATCTCCTCTGTCAGG-3’. The second TALEN recognition sequences were: Left TALEN 5’-AGACAACATCCCCTTCA-3’, right TALEN 5’-GTGTGACACAAAGCTTT-3’ with a 15-bp spacer 5’-CCGCCACCATGGTAA-3’. The zebrafish *oca2* TALEN recognition sequences were: Left TALEN 5’-AGTTCCTCTGTCCAT-3’, right TALEN 5’-TTCCACCGTCACGTAC-3’ with a 15-bp spacer 5’-GACTAACCAGTCGCT-3’ containing a *Bsr*I restriction enzyme site (underlined).

TALENs were constructed as described previously using the Golden Gate TALEN kit (Addgene) [38,44]. Assembled TALENs were cloned into the pT3TS-GoldyTALEN backbone for transcription [38]. mRNA was transcribed using the mMessage mMachine T3 Transcription Kit (Life Technologies) and purified using the RNeasy MinElute Cleanup Kit (Qiagen).

Eggs were collected immediately following spawning and 1-cell embryos were sorted from these clutches for injection. 1 to 2-cell embryos were injected with TALEN mRNA for individual TALEN pairs, or injected with both *oca2* exon 21 TALEN pairs. 400–800 pg of total TALEN mRNA was injected per embryo to achieve efficient mutation rates from injected TALENs in *Astyanax*. 50 pg of total TALEN mRNA was injected per embryo in zebrafish.

### Genotyping TALEN-induced mutations

Embryos were collected 12–48 hours after injection for genotyping. A portion of the embryos from both *mc1r* and *oca2* exon 9 injections of 800 pg of mRNA had morphological defects including bent bodies. For these injections half normal and half bent embryos were genotyped. DNA extraction was performed by placing embryos in 50 mM NaOH and incubating at 95°C for 30 minutes, followed by cooling to 4°C and adding 1/10^th^ volume of 1 M Tris-HCL pH 8 [45]. Regions around the TALEN recognition site were amplified by PCR using the following primers: *mc1r* forward primer 5’-ACCCCAGACCCCTCCTTT-3’, *mc1r* reverse primer 5’-TAGAGCCCGGCAGTGAATAC-3’, *oca2* exon 9 forward primer 5’-AAGTAAACAACAAATTACAATGTCAAA-3’, *oca2* exon 9 reverse primer 5’-GACTCCAGCCAGGATCACTC-3’, *oca2* exon 21 forward primer 5’-GAGAGTTTACCCAAAGCAGAGTG-3’, *oca2* exon 21 reverse primer 5’-ACCTGATATGCAACGCTCAA-3’, zebrafish *oca2* forward primer 5’-GGACCATACCACTGCACTCC-3’, zebrafish *oca2* reverse primer 5’-AGGATCAGACCAGCGATGAG-3’. PCRs for *mc1r* and *oca2* exon 9 and zebrafish *oca2* were divided in half, and one half was subjected to restriction enzyme digest. Restriction enzyme (RE) resistant bands represented alleles with TALEN-induced mutations. The *oca2* exon 21 TALENs were genotyped by looking for deletions in the region amplified by PCR by surveying for PCR products that were approximately 100 bp smaller than the wild type PCR product.

The percentage mutation rate was calculated for the *Astyanax mexicanus* and zebrafish genes by determining the percentage of uncut product (*mc1r* and *oca2* exon 9 and zebrafish *oca2*) or the percentage of deletion allele (*oca2* exon 21) from each PCR product. Images of gels (JPEGs) were analyzed in Fiji [46] using the gel analysis tools. Each band’s intensity value was calculated, and corrected for size of the band by dividing by the size of the band. The total intensity was calculated per lane by averaging the intensities per base pair of the wild type bands and adding that to the intensity per base pair of the mutant allele. The percentage of mutant alleles was calculated by dividing the corrected intensity of the mutant band by the corrected total intensity.

To confirm that there were TALEN-induced mutations in *Astyanax mexicanus* in this region, *mc1r* and *oca2* exon 9 restriction enzyme resistant bands and the 100-bp smaller band from the *oca2* exon 21 PCR were gel purified and cloned using a TOPO TA cloning kit (Life Technologies). Four to five clones were sequenced from one individual for each of the regions. Sequences were aligned to the wild type sequence with ApE (http://biologylabs.utah.edu/jorgensen/wayned/ape/). This method was also used to sequence each of these regions from two uninjected sibling fish, which confirmed that the TALEN binding sites and spacer regions in the siblings were identical to the sequences obtained from the cavefish genome.

The percentage of surviving embryos in *Astyanax mexicanus* was assessed for three trials of injections at 9–12 hours post injection. Embryos were collected from multiple breeding tanks, and the single celled embryos were sorted away from the rest of the embryos. The 1 to 2-cell embryos were injected, and the rest of the embryos were kept as control embryos. The percentage survival of embryos shown in Table 1 is for all of the embryos pooled from the three trials. However, there was variable survival between different clutches of fish. The survival for each clutch across different trials is shown in S1 Table. Note that for the *mc1r* and the *oca2* exon 9 injections, assessment of survival was done at a different time point from the assessment of injection efficiency. Also note that at the time of the first *oca2* exon 21 collection, there were very few embryos, and therefore all embryos were collected and injected, and no control embryos were assessed. We performed a Kruskal-Wallis one-way analysis of variance test to test for differences in survival among groups of control and injected fish and did not find any significant differences in survival among groups (H = 3.9, df = 5, p = 0.56). We also performed pairwise Pearson’s chi-square tests using total number of surviving and dead embryos for each condition (S2 Table). All statistics were performed using R [47]. A Bonferroni correction was applied to all chi-square p-values to correct for multiple testing.

**Table 1:** Percentage of surviving embryos at 9–12 hours post injection.

TALEN	Total mRNA concentration	Number of live embryos	Total number of embryos	Percentage survival
Control	0	1482	2245	66
Oca2 exon 9	400 pg	174	332	52
Oca2 exon 9	800 pg	226	364	62
Mc1r	400 pg	126	238	53
Mc1r	800 pg	201	346	58
Oca2 exon 21	400 pg	184	340	54

The number of total and surviving embryos were calculated for control and injected embryos in the morning after three sets of injections for each TALEN pair. mRNA is the total amount of mRNA injected per embryo. Percentage survival was calculated by dividing the number of living embryos by the total number of embryos. Survival of embryos for each trial can be found in S1 Table.

Transmission to the F_1_ generation in *Astyanax mexicanus* was determined by crossing four *oca2* exon 9 400 pg-injected adult F_0_ individuals to wildtype surface fish. Embryos were collected and pooled into groups of 10. Between 2 and 5 pools were assessed for each of the four crosses performed. DNA and genotyping was performed as described above. One of the 4 F_0_ fish transmitted mutant alleles. Percentage of mutant alleles was calculated as described above for F_1_ fish obtained from this cross, and the average percentage was calculated across the four pools sampled. TA cloning and sequencing of mutant alleles was also performed as described above.

### L-DOPA assays

L-DOPA assays were performed on scales as described previously [48]. Briefly, fish were anesthetized and scales were removed. Tissue was imaged and then placed in 4% formaldehyde for 1 hour at room temperature, washed in deionized water, rinsed once with the L-DOPA solution, stained with L-DOPA (Sigma-Aldrich), rinsed with deionized water, and imaged again. L-DOPA assays were performed on 10 surface fish scales, 10 Pachón cavefish scales from 6 fish and 9 scales from the non-melanin pigmented patches of 5 400 pg *oca2* exon 9 injected F_0_ individuals. One of these 9 scales had one melanophore visible before treatment with L-DOPA.

## Results

### TALEN design and assessment

**Fig 1:**
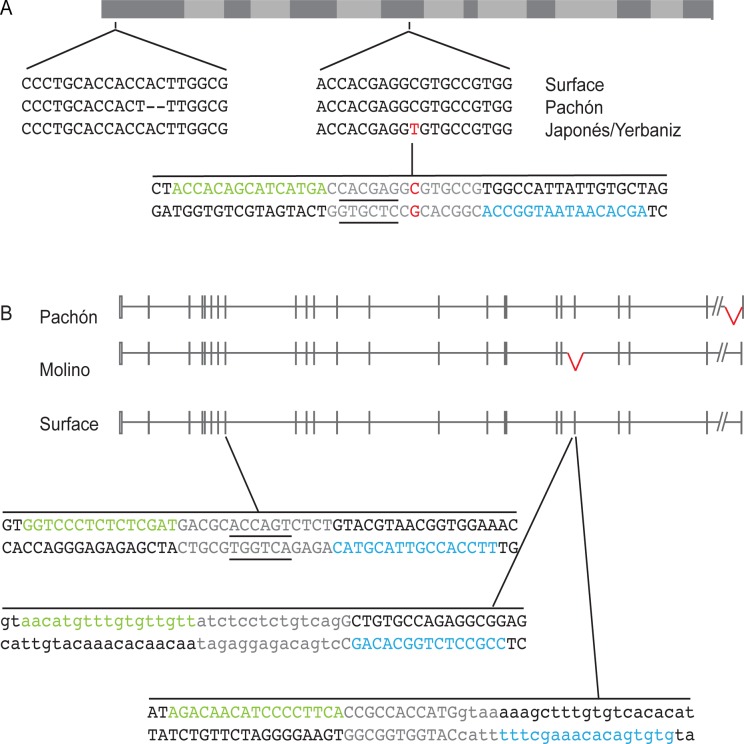
*mc1r* and *oca2* TALEN targeting design. A. Diagram of the *mc1r* annotated coding sequence with the location and sequences of the Pachón, Japonés and Yerbaniz mutations highlighted. The transmembrane domains are indicated in light gray. The two base pair deletion in Pachón is indicated by dashed lines. The single nucleotide change in the Japonés and Yerbaniz populations is in red. The sequence targeted by the TALEN is indicated below. The TALEN 1 binding site is in green and the TALEN 2 binding site is in blue. The spacer region is gray. The underlined sequence is the *Bss*SI restriction enzyme recognition sequence used for genotyping. The gene structure is based on the *Astyanax* genome sequence database and Gross et al. 2009 [19]. B. Diagram of the *oca2* gene. Boxes indicate exons and lines indicate introns. The empty boxes are UTR and the closed boxes are coding sequence. The slanted lines indicate a region of the genome left out because the distance is unknown. Note that the small scale of the figure resulted in some exons not being to scale and the size of some introns being so small that they appear to be one continuous exon. The exon 24 Pachón and the exon 21 Molino deletions are indicated in red. The amount of intronic sequence deleted in these populations is currently unknown. TALENs were designed targeting exon 9 and the either end of exon 21. The TALEN 1 binding sites are in green and the TALEN 2 binding sites are in blue. The spacer regions are gray. The underlined sequence in the exon 9 TALEN is the *Bsr*I restriction enzyme recognition sequence used for genotyping. The exon sequence is capitalized and the intronic sequence in the two exon 21 TALENs is lower case. The genomic structure is based on the *Astyanax* genome sequence database and Protas et al. 2006 [17]. Both gene structures were generated using http://wormweb.org/exonintron and then modified.

TALENs were designed to two genes that contain coding mutations in cavefish and lie under QTL for pigmentation, *mc1r* and *oca2*. Different cavefish populations have two different coding mutations in *mc1r*, a two base pair deletion that results in a frameshift and an early stop codon in the Pachón cavefish population, and a missense mutation in the Japonés and Yerbaniz cavefish populations [19]. As both of these mutations are thought to result in loss of function of *mc1r*, a TALEN was designed targeting the region around the missense mutation. TALENs are designed in pairs surrounding a spacer region within which the dimerized *FokI* will create a double stranded break. The *mc1r* TALEN was designed to a spacer region that contains a *Bss*SI restriction enzyme (RE) site. If an indel is created after repair of a TALEN cut at this site, the RE site is destroyed, creating a restriction fragment length polymorphism and an easy means of assessing TALEN efficiency (Fig. 1A).

Two cavefish populations contain coding deletions in *oca2* when compared to the surface population. Exon 21 is deleted in Molino fish, and a portion of exon 24 is deleted in Pachón cavefish [17]. Three TALENs were designed to the *oca2* surface sequence. One TALEN was designed to target exon 9, with a spacer region that contains a *Bsr*I RE site (Fig. 1B). We designed two TALENs surrounding exon 21 to generate cavefish-like mutations by removing all of exon 21. These TALENs were designed at the exon-intron boundaries of exon 21 (Fig. 1B).

**Fig 2:**
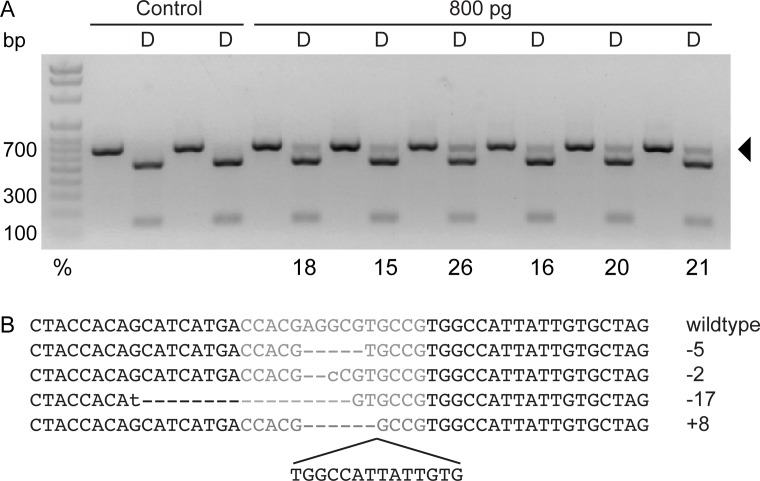
Analysis of mutagenesis in *mc1r* TALEN F_0_ fish. A. Genotyping gel of uninjected control and 800 pg-injected embryos. A portion of the *mc1r* genomic region was amplified by PCR from individual embryos, and half of the PCR product was digested (D) with *Bss*SI. Wild type DNA digests completely with *Bss*SI whereas alleles with mutations induced by the TALEN pair are resistant to restriction digest, indicated by the arrow. The percentage of mutant alleles is indicated below the band. Note that some mutant alleles may not have lost the restriction site and may still be sensitive to restriction digest. B. Sequence of a wild type surface fish and sequence of four clones from the restriction enzyme resistant band from a *mc1r*-injected individual. The dashed lines indicate missing nucleotides, the lower case letters indicate mismatches, and the sequence below the last clone is additional nucleotide sequence. The total number of base pairs more or less than the wild type sequence is indicated to the right of each clone.

Injections of 800 pg of *mc1r* TALEN mRNA resulted in RE resistance ranging from 15–26% (Fig. 2A). We found significant differences in survival between TALEN-injected and uninjected fish (S2 Table), with fewer injected embryos surviving compared to uninjected (Table 1). To confirm the presence of indels at this locus, the RE-resistant band from one individual was cloned, and four clones were sequenced. All four of these clones contained indels within the TALEN cut site (Fig. 2B).

**Fig 3:**
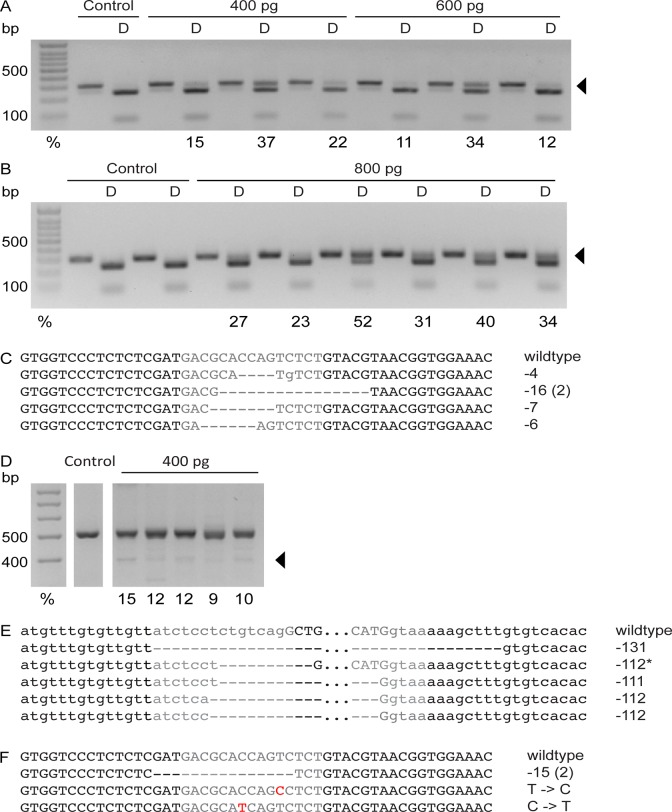
Analysis of mutagenesis in *oca2* TALEN-injected F_0_ fish. A. Genotyping gel of uninjected control and embryos injected with 400 pg and 600 pg of the *oca2* TALEN targeting exon 9. A portion of the *oca2* genomic region was amplified by PCR around exon 9 from 10 individual control embryos or individual injected embryos, and half of the PCR product was digested (D) with *Bsr*I. Wild type DNA digests completely with *Bsr*I whereas alleles with mutations induced by the TALEN pair are resistant to restriction digest, indicated by the arrow. The percentage of mutant alleles is indicated below the band. Note that some mutant alleles may not have lost the restriction site, and may still be sensitive to restriction digest. B. Genotyping gel of uninjected control and embryos injected with 800 pg of the *oca2* TALEN targeting exon 9. A portion of the *oca2* genomic region was amplified by PCR around exon 9 from individual control embryos or individual injected embryos and half of the PCR product was digested (D) with *Bsr*I. Wild type DNA digests completely with *Bsr*I, whereas alleles with mutations induced by the TALEN pair are resistant to restriction digestion, as indicated by the arrow. The percentage of mutant alleles is indicated below the band. Note that some mutant alleles may not have lost the restriction site, and may still be sensitive to restriction digest. C. Sequence of a wild type surface fish exon 9 and of five clones from the restriction enzyme resistant band from an *oca2*-injected individual. The dashed lines indicate missing nucleotides, and the lower case letters indicate mismatches. The total number of base pairs less than the wild type sequence is indicated to the right of each clone. Note that two of the five clones were identical. D. Genotyping gel of uninjected control and embryos injected with 400 pg total mRNA of the *oca2* TALENs targeting either side of exon 21. A portion of the *oca2* genomic region was amplified by PCR around exon 21 from 10 pooled control embryos or individual injected embryos. A band approximately 100 base pairs lower than the wild type band (arrow) indicates mutant alleles in injected embryos. The percentage of mutant alleles is indicated below the band. E. Sequence of a wild type surface fish around exon 21 and of five clones from 100 base pair smaller band from an *oca2*-injected individual. The dashed lines indicate missing nucleotides. The lower case letters are intron sequence, and the upper case letters are exon sequence. Sequence between the TALEN pairs is not shown and is indicated by the three dots. The total number of base pairs less than the wild type sequence is indicated to the right of each clone. Note that the starred clone contains a large deletion (321 bp) and an insertion (209 bp). F. Sequence of a wild type surface fish exon 9 and of four mutant clones from the restriction enzyme resistant band from pools of F_1_s from a cross between an *oca2* exon 9 400 pg-injected F_0_ individual and a wildtype surface fish. The dashed lines indicate missing nucleotides, and the red letters indicate mismatches. The total number of base pairs less than the wild type or the mutation compared to wildtype sequence is indicated to the right of each clone. Note that two of the four clones were identical.

The *oca2* exon 9 TALEN pair was injected at concentrations of 400, 600 and 800 pg mRNA (Fig. 3A and B). Fewer injected fish survived than uninjected fish (Table 1 and S2 Table). Increasing concentrations of mRNA increased efficiency. The efficiency in 400 pg-injected fish ranged from 15–37%, while the efficiency in 800 pg-injected fish ranged from 23–52% (Fig. 3A ad B). Five clones were sequenced from RE-resistant bands from one 400 pg-injected individual, and all five clones contained deletions (Fig. 3C).

The two *oca2* exon 21 TALEN pairs were coinjected into embryos to generate a deletion of exon 21. Similar to the other TALEN pairs, injection affected survival (Table 1 and S2 Table). PCR amplification of the region containing the deletion was performed, and bands that were approximately 100 bp smaller than the wild type PCR amplicon were quantified to determine TALEN efficiency. The efficiency of these injections ranged from 9–15% (Fig. 3D). The smaller band was cloned from one individual, and five clones were sequenced. All of the clones contained deletions between the two TALEN pairs, and four out of the five clones contained deletions greater than 100 bp (Fig. 3E). One of the five clones contained a larger deletion and a portion of the deleted sequence was duplicated and inserted within the deletion.

To analyze transmission of TALEN-induced mutations, *oca2* exon 9 400 pg-injected F0 fish were crossed to wildtype surface fish and pools of F1 embryos from these crosses were analyzed. Of the four F_0_ animals that produced offspring, one individual produced F_1_ embryos with restriction digest resistant alleles. 11.5% of the total alleles were restriction digest resistant in this cross, and sequencing and analysis of four clones containing mutant alleles revealed point mutations within the restriction site and a fifteen base pair deletion (Fig. 3F). These preliminary results suggest that obtaining germline transmitting mutant alleles after TALEN injection is feasible in *Astyanax mexicanus*.

### Assessment of *oca2* in TALEN-injected F_0_ fish

TALEN injections in other species have been shown to result in biallelic mutations (for example [38]). This means that, in principle, cell-autonomous phenotypes may be able to be assessed in F_0_ fish that are mosaic for these biallelic mutations. Mutations in *mc1r* are hypothesized to cause a reduction in pigmentation [19]. We did not observe any obvious differences in pigmentation in *mc1r*-TALEN injected fish compared to uninjected sibling fish. Loss of *oca2* results in albinism in a number of species (for example [49,50]), and *oca2* lies under the QTL peak for albinism in crosses between surface and albino cave *Astyanax mexicanus* [17]. As albinism is an obvious trait, we assessed fish injected with TALENs targeted to *oca2* exon 9 for evidence of mosaic absence of melanin pigmentation.

**Fig 4:**
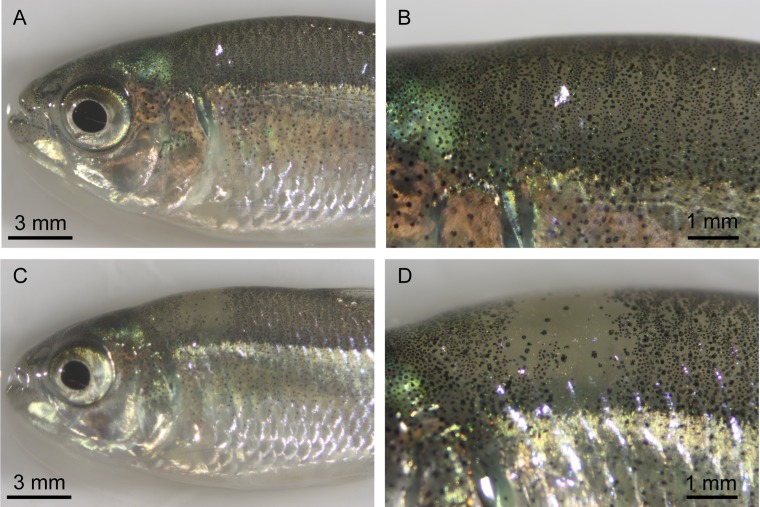
Analysis of pigmentation in *oca2*-injected F0s. A. Pigmentation in an uninjected surface fish. B. Close up of the dorsal region of the uninjected surface fish from A. C. Pigmentation in a 400 pg *oca2* exon 9 injected F0 surface fish. D. Close up of pigmentation patch lacking melanin pigmentation from panel C.

Larval injected fish did not display obvious differences in phenotype compared to uninjected control fish. However, juvenile 400 pg-injected fish were variegated in appearance, compared to the more even distribution of melanic pigment observed in wild type fish (Fig. 4A-D). Of our injected fish surviving to adulthood, 7 of the 12 400 pg-injected fish, 0 of the 5 600 pg-injected fish, and 0 of the 5 800 pg-injected fish had albino patches. The number of albino patches observed varied from one patch to eight patches per fish. None of the 15 uninjected sibling fish, the 3 o*ca2* exon 21-injected fish, or the 4 *mc1r*-injected fish contained similar albino patches. We examined the lighter colored patches under the microscope and observed a lack of melanin-producing melanophores in the regions that were lighter in appearance, suggesting that these patches are the result of biallelic TALEN-induced mutations in *oca2*.

**Fig 5:**
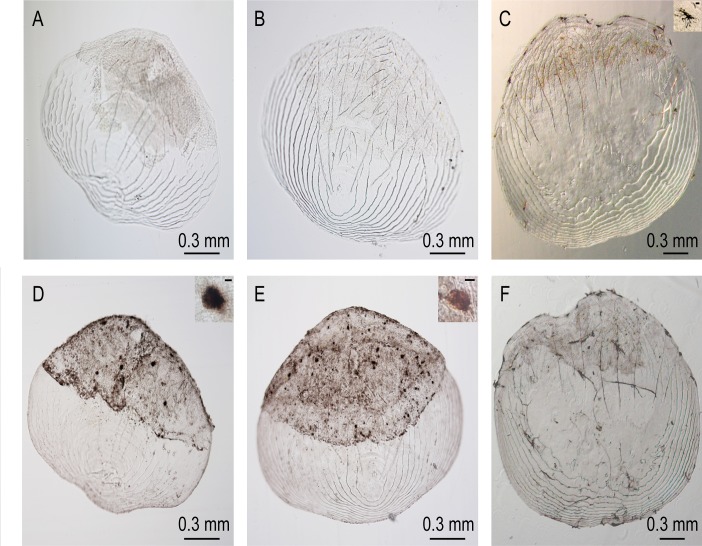
L-DOPA treated scales. A. A Pachón cavefish scale. B. A 400 pg *oca2* exon 9 injected scale from a non-melanin pigmented patch. C. An uninjected surface fish scale. D. The Pachón cavefish scale from A following treatment with L-DOPA. E. The 400 pg *oca2* exon 9 injected scale from B following treatment with L-DOPA. F. The uninjected surface fish scale from C following treatment with L-DOPA. Insets are close up pictures of a melanophore (in C) and melanin-producing cells (D and E). The scale bar in inset C is 20 uM. The scale bars in insets D and E are 10 uM. Note that dark patches on the unstained scales are tissue and that dark spots on B are bubbles or particles, not melanophores.

Mutations in *oca2* result in tyrosinase-positive albinism in humans [51]. The *oca2* gene encodes a putative 12-pass melanosome membrane protein [49,52]. Melanin synthesis in cavefish is disrupted at the first step in melanin synthesis, the conversion L-tyrosine to L-DOPA [53]. Albino cavefish contain pigment cell precursors lacking melanin, but which are capable of producing this pigment in lightly fixed specimens supplemented with L-DOPA [53]. We removed scales from albino cavefish and from non-melanin pigmented patches in *oca2* exon 9 TALEN-injected F_0_ fish, fixed them and then treated them with L-DOPA to determine if they contained cells capable of producing melanin pigment. Scales from albino fish and injected fish did not contain melanin pigmentation (Fig. 5A and B) with the exception of one scale from an injected fish, which contained one melanophore. This is in contrast to surface fish scales, which contain melanophores producing melanin (Fig. 5C). After treatment with L-DOPA, cells previously containing no melanin within scales from albino cavefish and surface fish produce melanin pigment [53]. We found that scales from injected fish and from albino cavefish produced melanin pigment after L-DOPA administration (9/9 injected scales, 9/10 albino cavefish scales, Fig. 5D and E). Additional cells produce pigment in an L-DOPA treated surface fish scale as well (Fig. 5F), as observed previously [53]. This confirms that the pigment-lacking patches in injected fish contain cells capable of producing melanin upon L-DOPA treatment, just as scales from albino cavefish do.

## Discussion

TALENs have been used for genome editing in an increasingly large number of model and non-model organisms [35–39,42]. We demonstrate here that TALENs can also be used to create mutations at specific loci in *Astyanax mexicanus*, an organism that has become a powerful model system for studying the genetic basis of evolution in an extreme environment, the cave.

Our results established that TALENs in *Astyanax mexicanus* can be highly efficient, with one TALEN pair generating up to 50% mutant alleles in individual embryos. Efficiency varied depending on the TALEN pair injected, similar to what is obtained with different TALEN pairs in zebrafish [38,54]. We also observed signs of toxicity that include bent bodies compared to control fish and we found significant differences in RNA toxicity as assessed by survival in injected compared to control fish (S2 Table). We did not find significant differences in survival as we increased the concentration of mRNA injected for individual TALEN pairs. However, this may be due to the high variability in survival between clutches of fish and between injections (S1 Table). It is not typical and it is difficult to assess effects of RNA toxicity from TALEN injection at late stages, for instance larval stages to adulthood, because embryos often appear normal if they make it through early development without any obvious defects in the embryonic axes. However, we cannot rule out that there are differences in survival to adulthood that are due to effects from TALEN injection beyond early stages of development. We had to use significantly more mRNA to induce mutations in *Astyanax mexicanus* when compared to zebrafish [38]. This may be due in part to the difference in size of the embryos, as zebrafish embryos are slightly smaller than *Astyanax mexicanus*. However, this size difference is unlikely to account for all of this disparity. It is possible that another method, such as CRISPRs, may result in more efficient mutation rates in *Astyanax mexicanus* with lower concentrations of total RNA.

In zebrafish, the frequencies of indels reported in genes targeted by TALENs in somatic tissue are similar to the frequencies transmitted through the germline [38,39,55]. Exceptions to this are genes required during embryogenesis [38]. This differs from rates of homologous recombination-mediated genome editing, which are much lower in germline transmission relative to somatic events [56]. In a preliminary screen of founder fish, we found that one of the four *oca2* exon 9 400 pg-injected F_0_ fish we crossed transmitted mutant alleles, as assessed by restriction digest and sequencing. The percentage of mutant alleles from this cross was 11.5%. As this cross was between an F_0_ individual and a wildtype surface fish, the percentage transmission (23%) was within the range of the percentage of somatic tissue mutated (15–37%) in 400 pg-injected fish. While it would be useful to identify additional transmitting F_0_ fish to get the range of the frequency of mutant allele transmission, our initial results indicate that this tool will be useful for isolating mutant lines of fish in the future for further genetic studies, even in situations were only a small number of founder fish can be raised to adulthood.

An advantage of the *Astyanax mexicanus* system for studying the genetic basis of evolution is that coding mutations have been identified in cavefish relative to surface fish for multiple candidate genes. To truly understand how an allele of a gene affects a trait, it will be useful to generate exact cave mutations, or cave-like mutations similar to alleles seen in cave populations. For example, two different types of mutations have been identified in *mc1r*, a two base pair deletion and a single base pair substitution. TALENs have been used in zebrafish to make precise mutations by injecting a TALEN pair with a single stranded oligo containing a mutation and getting homology directed repair off of the oligo [38]. This type of experiment could be performed using TALENs in *Astyanax mexicanus* to generate cave alleles, for example, of these two *mc1r* mutations, to test their effects on pigmentation. This also illustrates an advantage of TALENs over CRISPRs, as TALENs can be targeted to nearly any site in the genome, which would be necessary for this type of experiment, whereas only limited sites can be targeted by CRISPRs, as they require a PAM sequence [57].

Cavefish coding mutations can be larger deletions than those obtained by injection of a single TALEN, such as those seen in *oca2*. In zebrafish, large deletions can be generated by injecting two TALEN pairs [58]. We have demonstrated here that injecting two TALENs together in *Astyanax mexicanus* can generate deletions of sufficient size to create cavefish-like alleles. This provides an additional method for making cave-like mutations that may be important for determining the role cavefish alleles play in generating cave traits.

TALENs can generate biallelic mutations in zebrafish, allowing for assessment of phenotypes in injected F_0_ populations [38]. We did not see any obvious phenotypic differences between *mc1r*-injected individuals and wild type fish by eye. This could be due to the relatively low mutation rates from this TALEN pair, due to the subtle phenotype thought to be the consequence of *mc1r* mutations, which is a reduction in the amount of melanin pigmentation rather than a complete loss of melanin, the nature of the allele(s) generated by the TALEN pair, or due to the small number of *mc1r*-injected individuals raised to adulthood. Further analysis of this phenotype, in additional F_0_ injected fish or in fish transmitting this allele through the germline, would clarify the functional consequence of loss of this gene in future work. Albinism in cavefish is a recessive trait [17]. We did not observe albino patches in the *oca2* exon 21 TALEN-injected individuals. This could be due to the low efficiency of mutation in these injections or due to the small number of individuals raised to adulthood, as for the *mc1r* locus. However, there are albino patches in F_0_ *oca2* exon 9 TALEN-injected individuals, suggesting that the cells in the albino patches are the result of biallelic mutations. It is unlikely that these patches are due to RNA toxicity or an off target effect as they are not observed in fish injected with the other TALEN pairs. Furthermore, similar patches are seen in zebrafish injected with a TALEN pair also targeting exon 9 of *oca2* (S1 Fig.). We verified that these patches in *Astyanax mexicanus* were indeed albino by demonstrating that tissue from these areas behaves similarly to albino cavefish tissue and produces melanin pigment following treatment with exogenous L-DOPA. This experiment supports the idea that these non-pigmented patches are due to mutations at the *oca2* locus rather than off target effects of TALENs, as they result in a similar phenotype to *oca2*-mutant tissue in albino cavefish. These experiments also support the hypothesis that loss-of-function *oca2* alleles in cavefish cause albinism in these fish, consistent with QTL mapping, the inability of cavefish alleles to rescue pigmentation in mammalian cell culture, and morpholino knockdown in surface fish [17,24].

A number of cave species have independently converged on albinism through loss of melanin pigmentation at the first step of the melanin synthesis pathway [48]. One hypothesis for this convergence is that excess L-tyrosine can be used for another, adaptive purpose, such as being converted into catecholamines and modifying behavior. This hypothesis is supported by morpholino studies in which *oca2* knockdown in surface fish resulted in an increase in the amount of dopamine [24]. When we raise germline-transmitting *oca2* mutant fish, we will have the ability to test this hypothesis directly by looking for changes in catecholamine levels and changes in behavior in subsequent generations that phenocopy cave behaviors. This work will provide us with powerful insights into why particular mutations rose to fixation in certain cave populations and the consequences of these mutations at a functional level.

We have successfully demonstrated that TALENs can be used to generate small and large mutations in the surface form of *Astyanax mexicanus*, a model for studying the evolution of cavefish traits. Founder fish transmitting mutant alleles can be identified, which will be essential for isolating lines of mutant fish for future studies. Furthermore, this technology can be used to assess phenotypes in F_0_ fish, even for a trait that is recessive. Using TALENs to generate lines of fish with targeted mutations will be an important advancement towards confirming candidate genes and characterizing their role in the evolution of cave-specific traits.

## Supporting Information

S1 Fig Pigmentation in *oca2* TALEN-injected zebrafish. A. Genotyping gel of a pool of 10 embryos injected with 50 pg zebrafish *oca2* TALEN. A portion of the *oca2* genomic region was amplified by PCR, and half of the PCR product was digested (D) with *Bsr*I. Wild type DNA digests completely with *Bsr*I whereas alleles with mutations induced by the TALEN pair are resistant to restriction digest, indicated by the arrow. The percentage of mutant alleles is indicated below the band. B. Uninjected adult zebrafish. C. Adult zebrafish injected with a TALEN targeting *oca2* exon 9.
(TIF)

S1 Table (PDF)

S2 Table (PDF)
