# Supplementary figures and images for: Genome Editing Using TALENs in Blind Mexican Cavefish, Astyanax mexicanus
Source: PLoS One. 2015 Mar 16;10(3):e0119370. doi: 10.1371/journal.pone.0119370 (PMC4361574; doi:10.1371/journal.pone.0119370)

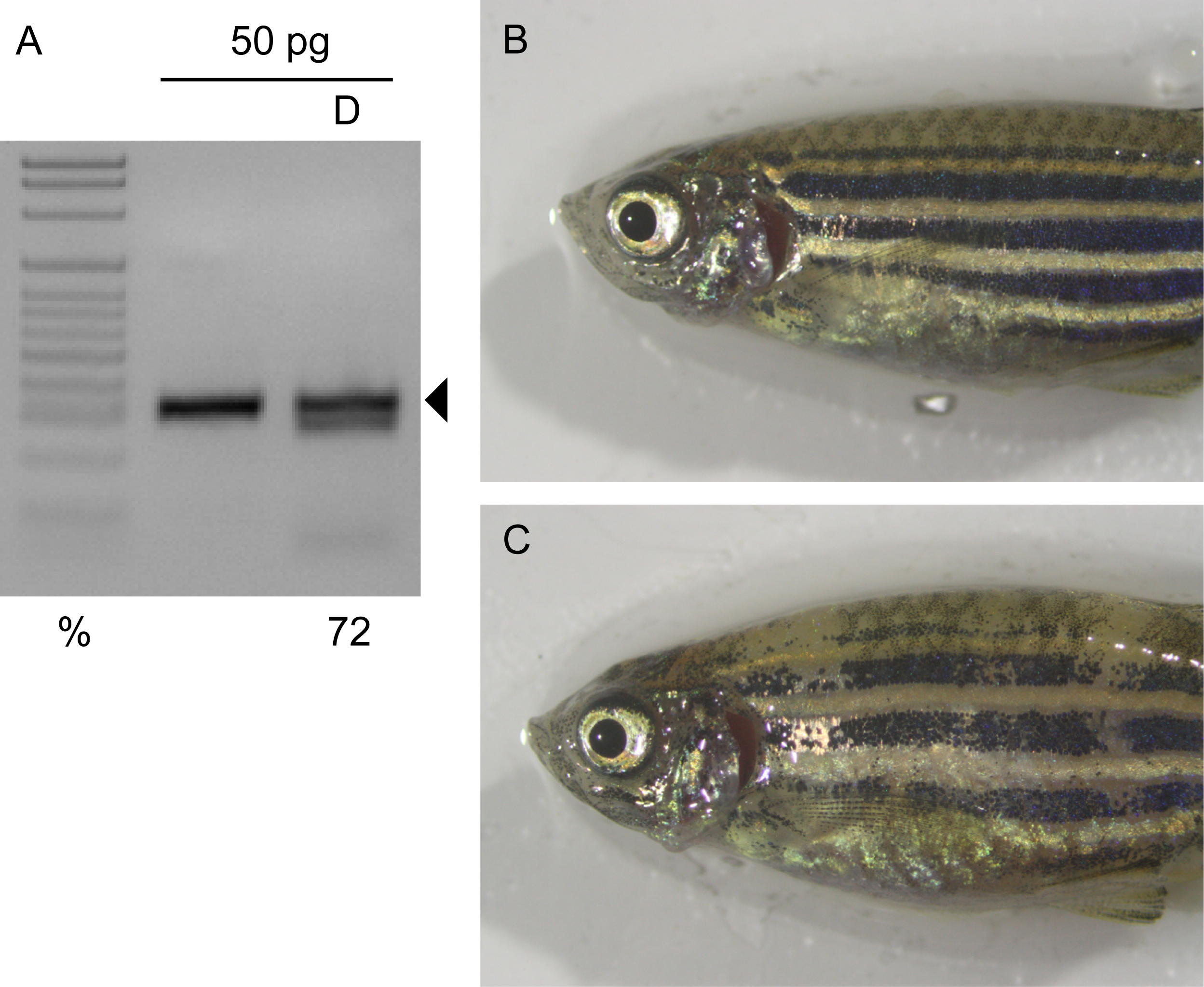

Supplement: S1 Fig — A. Genotyping gel of a pool of 10 embryos injected with 50 pg zebrafish oca2 TALEN. A portion of the oca2 genomic region was amplified by PCR, and half of the PCR product was digested (D) with BsrI. Wild type DNA digests completely with BsrI whereas alleles with mutations induced by the TALEN pair are resistant to restriction digest, indicated by the arrow. The percentage of mutant alleles is indicated below the band. B. Uninjected adult zebrafish. C. Adult zebrafish injected with a TALEN targeting oca2 exon 9. (TIF) [file pone.0119370.s001.tif]
