# Supplementary material for: Genome Editing Using TALENs in Blind Mexican Cavefish, Astyanax mexicanus
Source: PLoS One. 2015 Mar 16;10(3):e0119370. doi: 10.1371/journal.pone.0119370 (PMC4361574; doi:10.1371/journal.pone.0119370)
Supplement: S1 Table — (PDF) [file pone.0119370.s002.pdf]

TALEN	Total mRNA concentration	Parental tank ID	Number of live embryos	Total number of embryos	Percentage survival	Trial
Control	0	19	24	41	59	1a
Control	0	27	168	300	56	1a
Control	0	18	174	194	90	1a
Total control	0	19+27+ 18	366	535	68	1a
Oca2 exon 9	400 pg	19	49	101	49	1a
Oca2 exon 9	800 pg	19+27	33	85	39	1a
Mc1r	400 pg	28	23	33	70	1a
Mc1r	800 pg	28	59	108	55	1a
Oca2 exon 21	400 pg		15	53	28	1b
Control	0	19	138	196	70	1c
Control	0	28	300	527	57	1c
Total control	0	19+28	438	723	61	1c
Oca2 exon 9	100 pg	28	30	55	55	1c
Oca2 exon 9	200 pg	19	46	68	68	1c
Control	0	30	19	28	68	2
Control	0	28+29	305	438	70	2
Total control	0	28+29+ 30	324	466	70	2
Oca2 exon 21	400 pg	29	63	140	45	2
Oca2 exon 9	400 pg	30	58	132	44	2
Oca2 exon 9	800 pg	28	73	103	71	2
Mc1r	400 pg	28	53	109	49	2
Mc1r	800 pg	28	56	96	58	2
Total control	0	30	354	521	68	3
Oca2 exon 21	400 pg	30	106	147	72	3
Oca2 exon 9	400 pg	30	67	99	68	3
Oca2 exon 9	800 pg	30	120	176	68	3
Mc1r	400 pg	30	50	96	52	3
Mc1r	800 pg	30	86	142	61	3

Supplemental Table 1. Percentage of surviving embryos at 9-12 hours post injection.

The number of total and surviving embryos were calculated for control and injected embryos in the morning after one set of injections. mRNA is the total amount of mRNA injected per embryo. Parental tank ID indicates which tank of parents the each set of embryos was collected from. Percentage survival was calculated by dividing the number of living embryos by the total number of embryos. We performed the survival experiments 3 times, and each trial is indicated by the Trial number. Note that the trial 1 of the injections was done on 3 different days. The different days are indicated by a-c in the Trial column. Note that the lower concentrations of *oca2* TALEN (100 pg and 200 pg) did not produce mutations (data not shown) or induce bent bodies, and as such, we only performed these injections once.
