# Supplementary material for: Genome Editing Using TALENs in Blind Mexican Cavefish, Astyanax mexicanus
Source: PLoS One. 2015 Mar 16;10(3):e0119370. doi: 10.1371/journal.pone.0119370 (PMC4361574; doi:10.1371/journal.pone.0119370)
Supplement: S2 Table — (PDF) [file pone.0119370.s003.pdf]

Comparison	χ^2	df	p-value
Control, Mc1r 400 pg	16.1	1	0.0004
Control, Mc1r 800 pg	8.3	1	0.028
Control, Oca2 exon 21 400 pg	18.2	1	0.0001
Control, Oca2 exon 9 400 pg	23.3	1	0.00001
Control, Oca2 exon 9 800 pg	2.1	1	1
Mc1r 400 pg, Mc1r 800 pg	1.5	1	1
Oca2 exon 9 400 pg, Oca2 exon 9 800 pg	6.7	1	0.069

Supplemental Table 2. Chi square statistics.

Chi-square tests were performed on total surviving and total dead embryos at 9-12 hours post fertilization, comparing different injected conditions to control or each other. χ^2 is the chi-square statistic, df is degrees freedom and p-value is the Bonferroni-corrected p-value.
